# Primary mediastinal liposarcoma: a case report

**DOI:** 10.1186/1752-1947-1-161

**Published:** 2007-11-30

**Authors:** Nikolaos Barbetakis, Georgios Samanidis, Elpida Samanidou, Efthimios Kirodimos, Anastasia Kiziridou, Theodoros Bischiniotis, Christodoulos Tsilikas

**Affiliations:** 1 Thoracic Surgery Department, Theagenio Cancer Hospital, Thessaloniki, Greece; 2 Pathology Department, Theagenio Cancer Hospital, Thessaloniki, Greece; 3 Cardiology Department, Theagenio Cancer Hospital, Thessaloniki, Greece

## Abstract

**Introduction:**

Liposarcoma is the most commonly diagnosed soft tissue sarcoma in adults and occurs predominantly in the lower limbs and retroperitoneum. Primary mediastinal liposarcomas are rare.

They are often asymptomatic and when growing to large size the presenting symptoms are related to direct invasion or compression of other thoracic organs such as the heart, great vessels and lung.

**Case presentation:**

A case of a 68-year-old man with primary mediastinal liposarcoma involving the diaphragm and pericardium and successfully managed by complete surgical excision is presented. The patient's postoperative course was uneventful with no evidence of recurrence 9 months after the operation.

**Conclusion:**

Surgical removal is the optimal treatment for a mediastinal liposarcoma. If the entire tumor can not be resected, surgical debulking often results in symptomatic relief. International literature has demonstrated that recurrent disease occurs and therefore a long-term careful follow up is required.

## Introduction

Primary mediastinal liposarcoma is an uncommon neoplasm of intrathoracic origin. A case of primary mediastinal liposarcoma involving the diaphragm and pericardium, which was successfully managed by complete surgical excision is presented here.

## Case presentation

**Figure 1:**
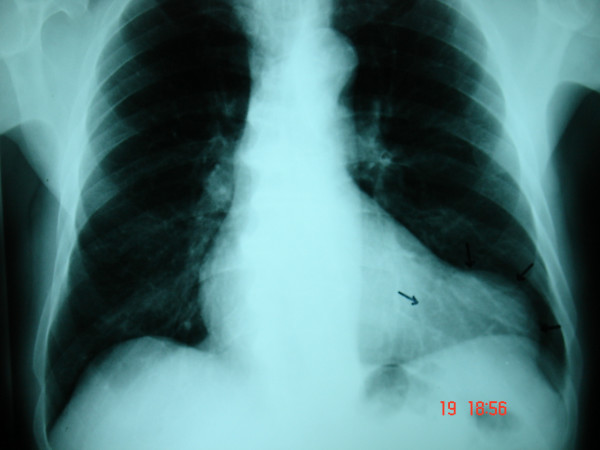
Chest x-ray revealed a soft tissue density mass on the left.

**Figure 2:**
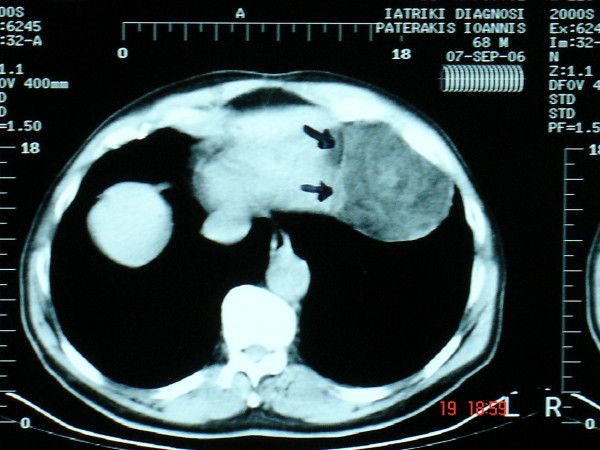
Computed tomography revealed an inhomogeneous fatty mass in the left hemithorax with invasive features to the heart and left hemidiaphragm.

A 68-year-old man who was otherwise well, presented with mild shortness of breath with 6 months duration and a recent onset of chest pain. Physical examination showed dullness on percussion and decreased breath sounds in the lower zone of the left lung. Laboratory data, respiratory function tests and arterial blood gas analyses were within the normal limits. Chest x-ray showed a large, well defined soft tissue mass in the anterior mediastinum (Figure 1). On computed tomography (CT), an inhomogeneous fatty mass in the left hemithorax showing invasive features to the heart and left hemidiaphragm was defined (Figure 2). Detection for distant metastases including bone scan, cranial and abdominal CTs showed no abnormal findings. Esophagoscopy and bronchoscopy revealed extrinsic compression effects, but no evidence of intraluminal tumor. Therefore surgical intervention was proposed. On the beginning the patient underwent a left anterolateral thoracotomy but due to diaphragmatic invasion, an abdominal extension of the incision was needed. A large, well-demarcated and slightly lobulated mass located in the left hemithorax showing invasive features to the heart and left hemidiaphragm was explored. The tumor was attached to the inferior pericardial wall causing significant compression to the heart. A pericardial incision was necessary to check possible myocardial infiltration. This manipulation revealed that the tumor was in close association with the left atrium and ventricle anteriorly but showed no invasion to these vital structures.

En bloc resection of the tumor with part of pericardium and the left hemidiaphragm was performed. The diaphragmatic defect was covered with bovine pericardium. The patient tolerated operation well and had an uneventful postoperative recovery. Nine months later he is free of disease and in a very good condition.

**Figure 3:**
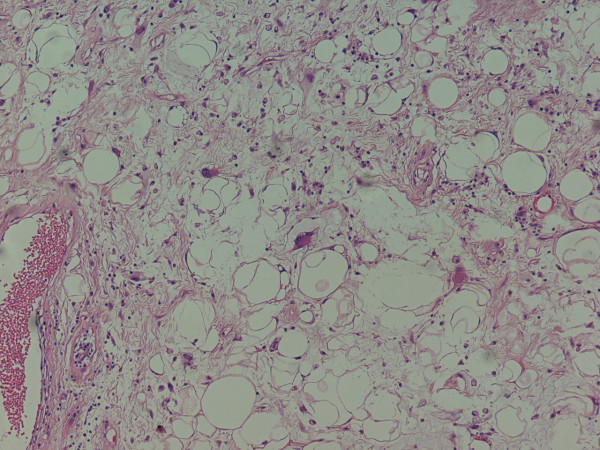
Photomicrograph shows fibrous bands containing atypical cells intermixed with fatty areas.

The entire tumor measured 9 × 7 × 5 cm in diameter and weighed 430 g in total. The mass was soft and pale yellow in color on cut section. The final pathologic diagnosis was well differentiated low grade liposarcoma (atypical lipomatous tumor, Figure 3).

## Discussion

Liposarcoma comprises approximately 1% of all malignancies and is the second most common soft tissue sarcoma in adults. The commonest site is the lower limb followed by retroperitoneum. Primary liposarcoma of the mediastinum is extremely rare, represents less than 1% of mediastinal tumors with less than 150 cases reported in the literature. It usually occurs in adults, with most cases occur in patients more than 40 years old [1,2]. Malignant liposarcoma develops more commonly in the posterior mediastinum. Liposarcoma of the anterior mediastinum is very rare and only a few cases have been reported in the international literature [3].

Mediastinal liposarcomas may extend into the pleural spaces and achieve a large size before detection. The presenting signs and symptoms are related to size and direct invasion of contiguous structures like the pericardium or superior vena cava [4]. Dyspnea, chest pain and tachypnea are the most common symptoms. Asymptomatic cases discovered by radiological imaging have also been reported [5]. In our case chest pain and mild shortness of breath were the main symptoms.

The predominant finding of mediastinal liposarcoma on conventional chest radiography usually, is a widened mediastinum. On CT, the appearance of mediastinal liposarcomas, as of liposarcomas located in any part of body, varies from a predominantly fat-containing mass to a solid mass. Low attenuation values between -50 and -150 Hounsfield Unit (HU) are consistent with a tissue composed of fat. Greater values are related to the necrosis, heterogenity and soft tissue component in liposarcomas. On magnetic resonance imaging (MRI), T1-weighted images show the fatty tissue with a high signal intensity, whereas the signal intensity diminishes in T2-weighted image. A differential diagnosis should be made between lipoma, thymolipoma, teratoma, lyphoma, germ cell tumor or even herniated peritoneal fat [6].

Pathologically four main types of liposarcomas have been described: myxoid, well differentiated, dedifferentiated and pleomorphic. Evans reported that survival in patients with dedifferentiated or pleomorphic liposarcomas was significantly shorter than in patients with myxoid or well differentiated liposarcomas [7]. Well differentiated low-grade liposarcomas, also known as atypical lipomatous tumors, have histologic features in many areas resembling mature adipose tissue. The cytoplasm of the atypical cells is usually indistinct or amorphous and occasional cytoplasmic vacuoles are noted [8]. Evans also reported that atypical lipomatous tumors may transform to dedifferentiated liposarcomas and usually do not metastasize [7].

## Conclusion

Surgical removal is the optimal treatment for a mediastinal liposarcoma, as in other sites. If the entire tumor can not be resected, surgical debulking often results in symptomatic relief. Radiotherapy and chemotherapy may be added as adjuncts to surgical excision but liposarcomas seem to have low sensitivity [9].

Recurrence is common in deep-seated liposarcomas and it becomes apparent within the first 6 months in most cases, but it may be delayed for 5 or 10 years following the initial excision [10]. Recurrence is related to the incomplete excision and tumor tissue left behind at the time of surgery. Therefore a close follow up is strongly recommended.

## Competing interests

The author(s) declare that they have no competing interests.

## Authors' contributions

NB was involved in the case directly (surgeon) and drafted part of the manuscript.

GS, ES, EK and TB took part in the care of the patient and contributed equally in carrying out the medical literature search and preparation of the manuscript.

AK was responsible for the pathology report.

CT participated in the care of the patient and had the supervision of this report. All authors approved the final manuscript.

## Consent

Written informed consent was obtained from the patient for publication of this case report and any accompanying images. A copy of the written consent is available for review by the Editor-in-Chief of this journal.
